# Silencing of miR-101 Prevents Cartilage Degradation by Regulating Extracellular Matrix–related Genes in a Rat Model of Osteoarthritis

**DOI:** 10.1038/mt.2015.61

**Published:** 2015-05-26

**Authors:** Linghui Dai, Xin Zhang, Xiaoqing Hu, Qiang Liu, Zhentao Man, Hongjie Huang, Qingyang Meng, Chunyan Zhou, Yingfang Ao

**Affiliations:** 1 Institute of Sports Medicine, Beijing Key Laboratory of Sports Injuries, Peking University Third Hospital, Beijing, P. R. China; 2 Department of Biochemistry and Molecular Biology, Peking University School of Basic Medical Sciences, Beijing, P. R. China

## Abstract

Osteoarthritis (OA) is a common, degenerative joint disease characterized by articular cartilage degradation. Currently, clinical trials based on microRNA therapy have been performed to treat various diseases. However, no treatment has been found for arthritis. This study investigated the functions of miR-101 in cartilage degradation *in vivo* and evaluated the feasibility of using miR-101 as a therapeutic agent for OA. Mono-iodoacetate-induced arthritis (MIA) rats were used as an animal model of OA. miR-101 mimic or miR-101 inhibitor was injected into the rats' knees to evaluate its effects on cartilage degradation. Cartilage degradation aggravated at 14 days after the injection of miR-101 mimic. By contrast, miR-101 silencing reduced cartilage degradation. Moreover, the administration of miR-101 mimic is sufficient to cause cartilage degradation in the normal cartilage of rats. By contrast, miR-101 inhibitor could prevent this change. Microarray and qPCR were used to investigate the different expressed genes after injecting miR-101 mimic and miR-101 inhibitor in the rats' articular cartilage. Several cartilage degradation-related genes were selected and validated to function in cartilage degradation with miR-101. Our results demonstrated the therapeutic effect of miR-101 inhibition on cartilage degradation in MIA rats by regulating several cartilage degradation–related genes.

## Introduction

Osteoarthritis (OA) is a common degenerative joint disease that causes joint pain, swelling, and even dysfunction. However, the treatment of OA is unsatisfactory: it is only limited to pain management. Clinically, nonsteroidal anti-inflammatory drugs, steroids, and hyaluronic acid cannot reverse the pathological process and can only relieve the symptoms of OA.^1,2,3^ In the late stages of OA, patients need joint replacement to improve their quality of life. OA is characterized by structural and biochemical changes in the articular cartilage, including progressive chondrocyte degradation and insufficient synthesis of its extracellular matrix (ECM).^4,5^ Cartilage degradation is irreversible and incurable because of its limited capacity for repair. Although several molecular mechanisms of cartilage degradation have already been characterized, the currently available information is still limited, and no effective methods can be used for OA prevention or treatment.^6,7,8^ Thus, further understanding of the molecular mechanism is important to investigate potential therapeutic targets.

Numerous studies suggested that microRNAs (miRNAs) have important functions in various diseases thereby possessing significant diagnostic and therapeutic potentials.^9,10,11,12^ miRNAs are small noncoding RNAs (containing about 22 nucleotides). miRNAs silence target mRNAs to induce target mRNA degradation or translational repression by binding to the 3′ untranslated regions.^13^ Currently, more than 100 clinical trials based on miRNA therapy have been performed to treat various diseases, such as cancers and cardiovascular disease (http://clinicaltrials.gov). However, treatment for arthritis has not been found, although several miRNAs have already been investigated and found to have important functions in OA,^14,15,16^ such as miR-140 (refs. 17,18) and miR-27b.^19^

A recent study has shown that miR-101 aggravates chondrocyte ECM degradation by directly targeting and regulating *Sox9* expression. Moreover, miR-101 silencing prevents IL-1β–induced chondrocyte ECM degradation *in vitro*.^20^ Another study found that miR-101 expression is higher in OA chondrocytes than that in normal chondrocytes.^21^ Therefore, studies should be conducted to determine whether miR-101 functions in animal OA pathogenesis *in vivo* and investigate the feasibility of using miR-101 as a potential therapeutic target.

In this study, mono-iodoacetate-induced arthritis (MIA) rats were used as an animal model of OA, because their histological and morphological changes in the articular cartilage are similar to those observed in human OA.^22,23,24^ The expression of miR-101 increased and that of *Sox9* decreased in the cartilage of MIA rats compared with those of normal rats. Subsequently, miR-101 mimic or inhibitor was injected into the knees of MIA rats as a therapeutic reagent. Cartilage degradation was aggravated at 7 and 14 days after the first injection of miR-101 mimic. By contrast, miR-101 inhibition reduced cartilage degradation *in vivo*. Further analysis by microarray showed that several cartilage degradation–related genes were regulated by miR-101, and cartilage-related cytokines in the joint synovial fluid were changed.

## Results

### Expression patterns of miR-101 and *Sox9* in the cartilage of MIA rats

**Figure 1:**
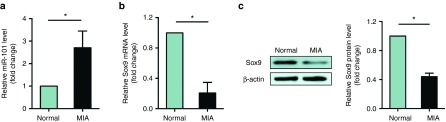
**Expression levels of miR-101 and *Sox9* in the cartilage of MIA rats.** The expression levels of (**a**) miR-101 and (**b**) *Sox9* relative to 18s RNA at 24 hours in the cartilage of MIA and normal rats were examined by qPCR. Three independent experiments were performed, each with different cartilage preparations of MIA rats. (**c**) *Sox9* protein level was examined by western blot. Normal cartilage was used as the control. *P < 0.05 indicates statistical significance.

To examine whether miR-101 participates in the cartilage degradation of MIA rats, the cartilage was harvested and analyzed after mono-iodoacetate injection at 24 hours. The expression of miR-101 significantly increased (**Figure 1a**). *Sox9*, a target of miR-101, significantly decreased at both the mRNA (**Figure 1b**) and protein levels (**Figure 1c**). These results were consistent with our previous findings.^20^

### Expression changes in miR-101 and *Sox9* after injecting Ad-miR-101 mimic and inhibitor in the cartilage of MIA rats

**Figure 2:**
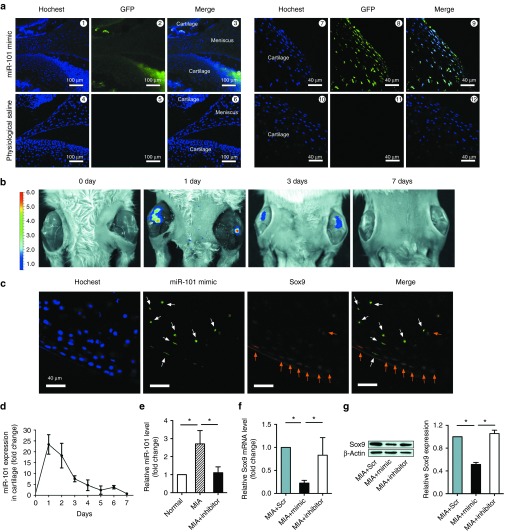
**Injected Ad-miR-101 mimic and inhibitor can penetrate into the cartilage and regulate *Sox9* expression.** (**a**) Images numbered 1 to 6 were observed at ×4 magnification. Images numbered 7 to 12 were obtained from the whole layer of cartilage and then observed at ×40 magnification. The nuclei were stained with Hoechst 33342. S indicates synovium. (**b**) The nuclei were stained with Hoechst 33342. Green fluorescence indicates the miR-101 mimic-expressing cells; red fluorescence indicates the *Sox9*-expressing cells. White arrows show the miR-101 mimic-expressing cells with low levels of *Sox9*; orange arrows show the *Sox9*-expressing cells with low levels of miR-101 mimic. miR-101 expression was examined using a (**c**) fluorescent imaging system and (**d,e**) qPCR after injecting Ad-miR-101 mimic. *Sox9* expression was examined with (**d**) real-time RT-PCR and (**e**) western blot 1 day after the injection of microRNA in MIA rats. The MIA+Scr group was used as the control. *P < 0.05 indicates statistical significance.

To test whether exogenous miR-101 can penetrate into the cartilage, frozen sections of rat knees were examined by confocal microscopy after the injection of green fluorescent protein (GFP)–tagged Ad (adenovirus)-miR-101 mimic, Ad-miR-101 inhibitor, or Ad-Scr (scramble). Under fluorescent microscopy, GFP was observed in the cartilage and synovium (**Figure 2a**, **1**–**6**), indicating that miR-101 could penetrate both the synovium and cartilage. The expression of miR-101 can penetrate into whole layer of cartilage (**Figure 2a**, **7**–**9**). The expression of miR-101 significantly increased 1 day after the injection and lasted for 6 days (**Figure 2b**,**d**). miR-101 expression increased in the cartilage of MIA rats and was reversed after the injection of Ad-miR-101 inhibitor. This result indicates that the inhibitory effect is via suppression of endogenous miR-101 (**Figure 2e**). Interestingly, the miR-101 mimic–expressing cells (GFP, green fluorescence) showed low *Sox9* expression (stained in red fluorescence), and low miR-101 mimic–expressing cells showed higher *Sox9* expression (**Figure 2c**), indicating that miR-101 regulated *Sox9*. The downregulated expression of *Sox9* in the cartilage of MIA rats was reversed after the injection of Ad-miR-101 inhibitor (**Figure 2f**,**g**). These results demonstrated that exogenous miR-101 mimic could penetrate into the cartilage, express miR-101, and regulate its target-*Sox9* expression.

### Effect of miR-101 treatment on cartilage degradation in MIA rats

MIA rats were used to evaluate the effects of exogenous miR-101 treatment on the degradation of articular cartilage.

**Figure 3:**
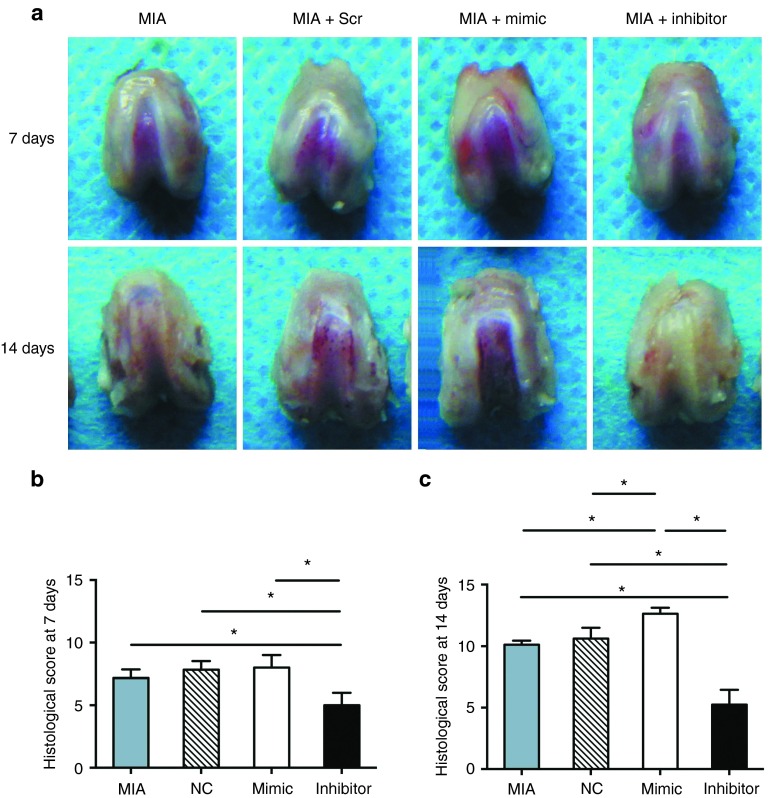
**Histological analysis of cartilage degradation in MIA rats with miRNA injection.** All experiments were performed 7 and 14 days after MIA injection. Eight rats in each group were obtained. (**a**) Representative image of each group. (**b,c**) Summary of the Mankin scores in the four groups 7 and 14 days after MIA injection. Higher scores indicate more serious cartilage degradation. Data are expressed as the mean ± SD. *P < 0.05 indicates statistical significance.

Macroscopically, no obvious differences in morphology were observed among the four groups after 7 days (**Figure 3a**). Aggravated cartilage degradation in the mimic group and less cartilage degradation in the inhibitor group were observed after 14 days (**Figure 3a**). Moreover, large subchondral bone exposures were presented in the mimic group in the trochlea of the femur. By contrast, a smooth cartilage surface was observed in the same area of the inhibitor group (**Figure 3a**).

A low histological score indicates less degradation according to the histological score system. The mimic group showed a higher score than the other three groups, indicating more cartilage damage after 14 days (**Figure 3c**). By contrast, the inhibitor group showed the lowest score among the four groups in the histological assessment at both 7 and 14 days. (**Figure 3b**,**c**).

**Figure 4:**
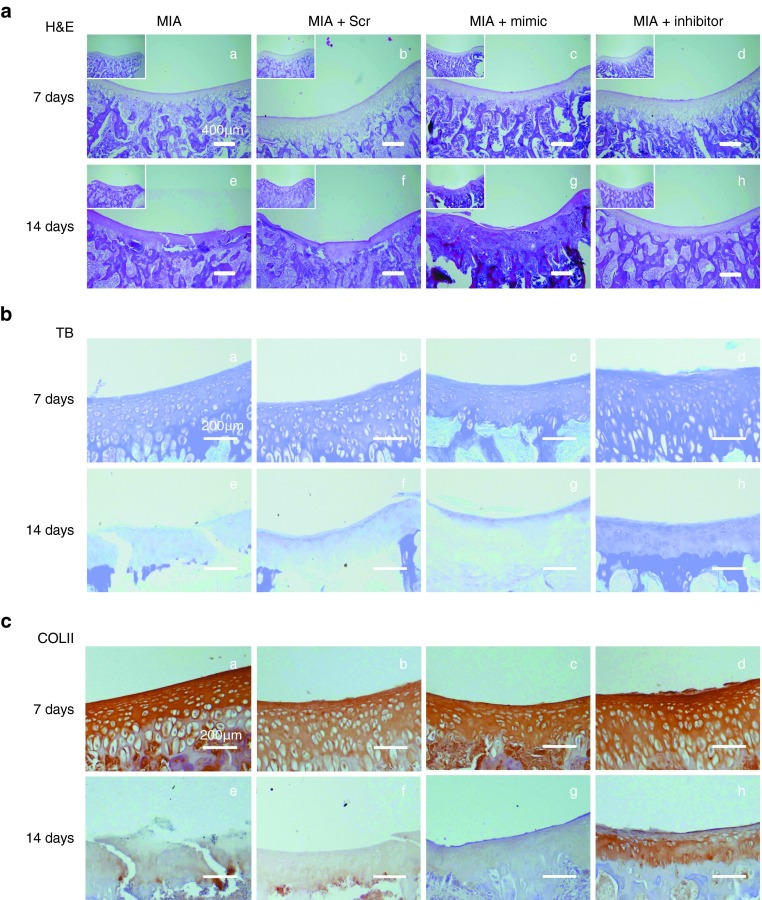
**Histological assessment of the cartilage in MIA rats with miR-101 mimic and inhibitor treatment.** Images in this figure are the representative images of each group. (**a**) HE staining images of each group. Original magnification is ×4. The image in the white frame is the same section at ×2 magnification. Bars = 400 μm. (**b**) Toluidine blue staining images of each group. Sections in the trochlea of the femur were stained with toluidine blue to assess cartilage damage and proteoglycan loss. Original magnification is ×10. Bars = 200 μm. (**c**) Immunohistochemical staining of collagen type II in each group. Original magnification is ×10. Bars = 200 μm.

Typical MIA-induced cartilage changes were observed in the MIA, Scr, and mimic groups through hematoxylin-eosin (HE) staining after 14 days (**Figure 4a**), including cartilage surface irregularities (**Figure 4a**,**e**–**g**) and clefts (**Figure 4a**,**e**), narrowing in the deep zone of calcified cartilage (**Figure 4a**,**e**–**g**), unclear tidemark (**Figure 4a**, **f**, and **g**), and severely damaged cartilage structure (**Figure 4a**, **f**, and **g**). By contrast, smooth surface, clear tidemark, and integrated cartilage structure were detected in the inhibitor group after 7 and 14 days (**Figure 4a**, **d**, and **h**).

Loss of ECM (lighter matrix staining in **Figure 4b**,**g**) was observed in the mimic group compared with that in the MIA and Scr groups. However, the ECM was densely stained with toluidine blue in the inhibitor group (**Figure 4b**,**h**).

In terms of collagen secretion, no significant differences in the immunohistochemical (IHC) staining of collagen type II were observed among the four groups after 7 days (**Figure 4c**,**a**–**d**). However, an evident positive staining was observed in the inhibitor group compared with other three groups after 14 days (**Figure 4c**,**h**).

The IHC staining of collagen type X was used to examine the hypertrophic chondrocytes. However, no difference was detected among the four groups at both 7 and 14 days (**Supplementary Figure S1A**). The expression levels of MMP-13 and ADAMTS-4 were also examined by IHC to assess the ECM degradation enzymes. However, no significant difference was noted among the four groups on both MMP-13 and ADAMTS-4 expression at 7 and 14 days (**Supplementary Figures S1B**,**C**).

### Mechanism study of miR-101 treatment on cartilage degradation

**Figure 5:**
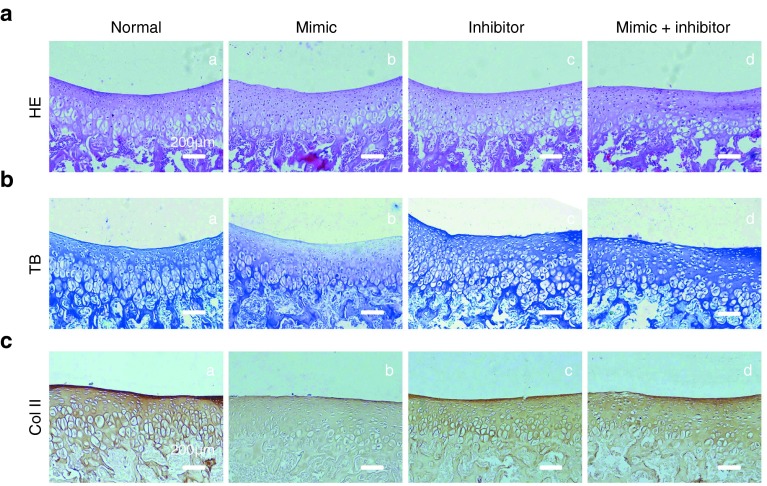
**Histological assessment of the cartilage in MIA rats with miR-101 mimic and inhibitor treatment.** Images in this figure are the representative images of each group. (**a**) HE staining images of each group. Original magnification is ×10. Bars = 200 μm. (**b**) Toluidine blue staining images of each group. Original magnification is ×10. Bars = 200 μm. (**c**) Immunohistochemical staining of collagen type II in each group. Original magnification is ×20. Bars = 200 μm.

Normal rats were divided into four groups: rats were injected with Ad-miR-101 mimic (mimic group), Ad-miR-101 inhibitor (inhibitor group), both Ad-miR-101 mimic and inhibitor (mimic+inhibitor group), and normal rats were used as control (normal group). These rats were used to assess whether the injection of miR-101 mimic is sufficient to cause cartilage degradation. Interestingly, we found that the secretions of aggrecan (**Figure 5b**) and collagen type II (**Figure 5c**) in the mimic group were significantly decreased among the four groups after the injection of Ad-miR-101 mimic at 14 days, although no difference was found in the structure of cartilage from HE staining (**Figure 5a**). However, no difference was detected in the IHC staining of collagen X, MMP-13, and ADAMTS-4 among the four groups (**Supplementary Figure S2**).

**Figure 6:**
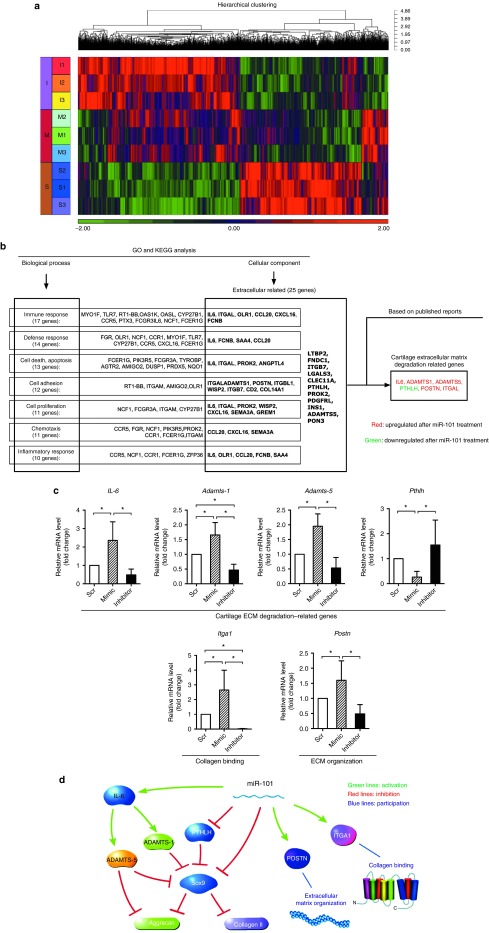
**Mechanism study of miR-101 on cartilage degradation *in vivo*.** (**a**) Hierarchical cluster analysis of gene expression (including miRNAs). Heat map shows the down- (green) or upregulation (red) of gene expression. Different samples were grouped using the hierarchical clustering algorithm. (**b**) The cartilage ECM degradation-related genes were screened from KEGG PATHWAY database, Gene Ontology analysis, and previous reports. (**c**) Cartilage ECM degradation-related gene expression was analyzed by qPCR. Gene expression was relative to 18s RNA. The Scr group was used as the control. *P < 0.05 indicates statistical significance. (**d**) Image shows the network regulation associated with cartilage degradation according to microarray data and previous reports. The relationships among these genes are supported by at least one reference from the literature, our data, or KEGG PATHWAY database and Gene Ontology analysis.

**Figure 7:**
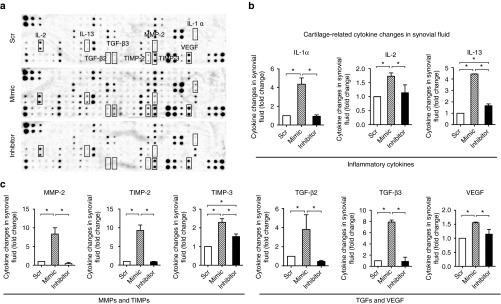
**Change in expression of cytokines in rat knee synovial fluid after miR-101 mimic and inhibitor injection.** The rats' knee joints were injected with Scr, miR-101 mimic, and miR-101 inhibitor. Cytokine expression in the synovial fluid was analyzed by antibody array (RayBio, AAR-BLM-1). (**a**) Representative images from each group. (**b,c**) Graph showing cartilage-related cytokine expression in both groups (other changed cytokines are shown in **Supplementary Table S2**), including inflammatory cytokines: IL-1α, IL-2, IL-13, matrix metalloproteinases and tissue inhibitors of matrix metalloproteinases, TGFs, and VEGF. Each expression level was normalized to that of the control group (Scr). Results are expressed as the mean ± SD (*n* = 4 rats per group). *P < 0.05, compared with the control group (Scr).

To study the mechanism of miR-101 on cartilage degradation, cartilage samples from the mimic group and inhibitor group at 1 day were examined by microarray. Rats injected with Ad-Scr mimic were used as controls. Hierarchical clustering showed the expression of mRNAs (including miRNAs) among the three groups (**Figure 6a**). We identified 106 genes (including 1 miRNA) that were differentially expressed in the mimic group versus the control group. Simultaneously, some of these genes were no significant changes when the inhibitor group was compared with the control group; some of these genes with an inverse expression to that of the mimic group (**Supplementary Table S1**). Eighty-five of these genes were upregulated, and 21 were downregulated at the presence of miR-101. Several cartilage-related molecules were detected, including *Il-6*, *Adamts-1*, *Adamts-5*, *pthlh*, *Postn*, and *Itga 1*. We further validated and analyzed the changes in rat cartilage among the three groups using qPCR. We found that *Il-6*, *Adamts-1*, *Adamts-5*, *Postn*, and *Itga 1* were upregulated, whereas *Pthlh* was downregulated by miR-101, which was consistent with the microarray data (**Figure 6c**). Moreover, *Pthlh* was predicted to be a target of miR-101 based on microRNA databases and luciferase assay (**Supplementary Figure S3**), suggesting that an interesting target of miR-101 needs further investigation. The network regulation image shows the regulation among these genes based on previous reports^25,26,27,28,29^ (**Figure 6d**). Furthermore, the changes in cytokines in the joint synovial fluid were analyzed using an antibody assay. The expression levels of cytokines were significantly increased after the injection of miR-101 mimic but decreased after the injection of miR-101 inhibitor compared with the control group (Scr; *P* < 0.05), including cartilage-related genes: IL-1α, IL-2, IL-13, MMP-2, TIMP-2, TIMP-3, TGF-β2, TGF-β3, VEGF (**Figure 7**), and other cytokines (**Supplementary Table S2**).

## Discussion

Many miRNAs, such as miR-140 (ref. 17), miR-34a,^30^ miR-27b,^19^ and other miRNAs, were found to function in cartilage degradation and considered as promising therapeutic targets of OA. Our previous report demonstrated that downregulated miR-101 expression prevented the IL-1β–induced ECM degradation in chondrocytes.^20^ However, limited information is available about the feasibility of using miRNA as a therapeutic target in the prevention of cartilage degradation in an animal model of OA.

This study investigated the feasibility of joint injection of miR-101 mimic or inhibitor to prevent cartilage degradation in MIA rats. To elucidate the feasibility of using miR-101 as a therapeutic target, several factors should be studied. First, the vectors used should be well considered for successful miRNA delivery after joint injection.^31,32,33^ In the present study, The Ad vector was used because of its high efficiency and ability to easily enter cells. Several studies have demonstrated that the Ad vector can penetrate into cartilage, and this Ad vector has been used to prevent experimental OA cartilage degradation by joint injection. In the present study, injected miR-101 was observed in both cartilage and synovium, indicating that miR-101 could directly penetrate cartilage or may do so through infected synovium secretions. Second, efficacy is a significant index for determining the feasibility of using a certain therapeutic target. Our previous study revealed that miR-101 targets *Sox9* in regulating ECM synthesis. In the present study, *Sox9* expression was regulated by injecting Ad-miR-101 mimic and inhibitor in the cartilage of MIA rats. Moreover, injecting miR-101 mimic worsened cartilage degradation, whereas silencing miR-101 reduced it in MIA rats. We found that the administration of miR-101 could sufficiently cause cartilage degradation, whereas miR-101 inhibitor caused recovery. The mechanism study demonstrated that miR-101 promoted the expression of several ECM degradation-related genes expression, *Il-6*, *Adamts-1*, *Adamts-5*, and *Postn* increased, but not *Mmp-13* and *Adamts-4*. We also found that *Pthlh* (parathyroid hormone-like hormone), which can affect *collagen type II* by regulating *Sox9*, is a potential target of miR-101 according to the miRNA target prediction databases and luciferase assay. We also found that IL-1α, IL-2, IL-13, MMP-2, TIMP-2, TIMP-3, TGF-β2, TGF-β3 and VEGF increased after the injection of miR-101 mimic and decreased after the injection of miR-101 inhibitor. These changes may influence cartilage degradation. Further investigations are needed to study and validate the crosslink between the changes in cartilage degradation–related genes and the changes of synovial fluid cytokines. Collectively, the injection of miR-101 affected not only the cartilage degradation–related genes but also the cytokine secretion of the synovium.

To our knowledge, this study is the first to evaluate the therapeutic effect of miR-101 inhibition on cartilage degradation in MIA rats. Although this preliminary study might provide an option for OA treatment, further studies are needed for improvement. For example, the locked nucleic acid–based miRNA delivery system is a potent therapeutic tool for developing miRNA mimic and inhibitor because it is highly stable for miRNA delivery, displays low toxicity in biological systems, and is easily made into drugs.^34,35^ In addition, clinical trials have been conducted using locked nucleic acid –modified oligonucleotides by target miRNA inhibition (http://www.clinicaltrials.gov/). However, whether or not locked nucleic acid–modified miR-101 inhibition can be used to prevent cartilage degradation in OA needs further investigation.

## Materials and Methods

***Induction of MIA and intra-articular injection of miRNA.*** All animal experimental protocols were approved by the Animal Care and Use Committee of Peking University and conformed to the protocols of the National Institutes of Health Guidelines. Sprague–Dawley rats weighing 150 g were used in this study. Both the knees of the rats were intra-articularly injected with 2 mg of mono-iodoacetate in 50 μl of physiological saline through the patellar ligament using a 26-gauge needle. Ad-miR-101 mimic and Ad-miR-101 inhibitor were injected 3 days (concentration = 1 × 109 pfu, 50 μl per knee, once per 3 days) after the injection of mono-iodoacetate and continued until the rats were sacrificed. The rats were divided into four groups, namely, the MIA group, MIA + Scr group (Scr group), MIA + mimic group (mimic group), and MIA + inhibitor group (inhibitor group). The rats in the MIA group were injected with physiological saline; the Scr group was injected with Ad-Scr; the mimic group was injected with Ad-miR-101 mimic; and the inhibitor group was injected with Ad-miR-101 inhibitor. The rats were sacrificed under anesthesia at 7 and 14 days after the first injection.

***Construction of miRNA Ad expression vectors.*** According to the sequences on miRbase (MI0000886), miR-101 mimic, inhibitor, and Scr were designed and synthesized by Genechem (Shanghai, China). The miR-101 mimic, inhibitor, and Scr templates were inserted into the miRNA expression plasmid pDC316-siRNA (kl626) (Genechem, Shanghai, China). Subsequently, pDC316-siRNA (kl626) was cotransfected with the infectious Ad genomic plasmid pBHG loxΔE1, 3 Cre (Microbix, Toronto, Canada) into 293 cells using Lipofectamine 2000. After the cotransfection of these DNAs, homologous recombination occurred to generate three recombinant Ads, namely, Ad-miR-101 mimic, Ad-miR-101 inhibitor, and Ad-miR-Scr.

***Histological evaluation.*** The distal portions of the rats' femurs (eight samples in each group) were cut off and fixed in 4% paraformaldehyde (pH 7.4) for 48 hours at 4 °C. The samples were then decalcified in a RapidCal Immuno decalcifier (BBC Biochemical, Washington, DC) for 2 days. The decalcified specimens were trimmed, dehydrated in a graded ethanol series, and embedded in paraffin. Sections (5 μm thick) were cut in the transverse plane through the central part of the femoral trochlea. The center of the section was used for histological evaluation to make it comparable among the different groups. Sections were stained with HE and toluidine blue. IHC analysis was performed with type II collagen antibody, type X collagen antibody (Abcam, Cambridge, UK), MMP-13 antibody, and ADAMTS-4 antibody (Santa Cruz Biotechnology, Santa Cruz, CA). The sections were examined by three investigators blinded to lesion treatments. A modified Mankin system was used to score the changes in the cartilage of the rats.^36^

***Immunofluorescence analysis.*** Sections obtained from the cartilage of MIA rats (1 day after injection) were rinsed in phosphate-buffered saline and then embedded in OCT compound (Sakura Finetek, Torrance, CA). Cryosections (8 μm thick) were mounted onto slides and then incubated with Hoechst 33342 for 5 minutes. After three phosphate-buffered saline washes, the slides were observed under a confocal microscope (FV 1000 Olympus IX-81; Olympus, Tokyo, Japan).

***RNA isolation and real-time quantitative PCR (qPCR) analysis.*** Cartilage were carefully harvested from the femoral condyle and tibial plateau of rats, a quick microscopic examination were performed to confirm the absence of contaminating non-cartilaginous tissue. Total RNA from the cartilage of MIA rats was extracted using TRIzol reagent (Invitrogen, Carlsbad, CA). Isolated RNA was reverse-transcribed using a commercial kit (Promega, Madison, WI), and qPCR analysis was performed using the ABI Step One plus qPCR System (ABI, Foster City, CA) with a SYBR Select Master Mix (ABI). The qPCR conditions were as follows: 50 °C for 2 minutes, 95 °C for 2 minutes, followed by 40 cycles of 95 °C for 15 seconds and 60 °C for 30 seconds. A dissociation stage was added at the end of the amplification procedure. No nonspecific amplification was determined by the dissolution curve. The PCR primers can be seen in **Supplementary Table S3**. To evaluate miR-101 expression, reverse transcription and PCR were performed using Bulge-Loop miRNA qPCR primer set (RiboBio, Guangdong, China) according to the manufacturer's instructions. The expression of these genes relative to 18s RNA and that of miR-101 relative to U6 (RiboBio) were determined using the 2^−ΔΔ^CT method.

***Protein isolation and western blot.*** Protein was extracted using lysis buffer (50 mmol/l Tris-HCl, pH 7.4, 150 mmol/l NaCl, 1% NP-40, and 0.1% sodium dodecyl sulfate), and its concentration was measured using the BCA protein assay kit (Pierce, Rockford, IL) with bovine serum albumin as the standard. Proteins were run on SDS–PAGE gels (10%) and electro-transferred onto nitrocellulose membranes at 4 °C for 2 hours. The blots were probed with anti-Sox9 (Millipore, Temecula, CA) at 1:4,000 dilution at 4 °C overnight and then incubated with horseradish peroxidase-conjugated secondary antibody (Santa Cruz, CA, 1: 1,000 dilutions) at room temperature for 1 hour. Proteins were detected by chemiluminescence according to the manufacturer's recommendations (ECL, Millipore). β-actin (Sigma, St. Louis, MO, 1:10,000 dilutions) was used as an internal control.

***Microarray and data analysis.*** The rats were injected with Ad-miR-101 mimic (M) or Ad-miR-101 inhibitor (I), and Ad-miR-scr was used as a negative control (N). Articular cartilage was isolated at 24 hours. Total RNA was extracted by Trizol and then purified with magnetic beads of Agencourt Ampure (APN 000132, Beckman Coulter, Fullerton, CA). Target preparation was carried out according to the GeneChip WT PLUS Reagent Kit. The samples were then hybridized to the Affymetrix Human Gene 1.0ST Array (Affymetrix, Santa Clara, CA). Following hybridization, microarrays were washed and stained with Streptavidin Phycoerythrin on Affymetrix Fluidics Station 450. Microarrays were scanned by the Affymetrix GeneChip Command Console, which is installed in GeneChip Scanner 3000. The data were analyzed with a Robust Multichip Analysis algorithm using default analysis settings, and global scaling was performed as a normalization method using Partek Genomics Suite 6.6 (Partek, Saint Louis, MO).

***Measurement of cytokine changes in the knee synovial fluid.*** The rats' knee joints were first injected with Ad-Scr, Ad-miR-101 mimic, and Ad-miR-101 inhibitor. Knee synovial fluid was harvested 24 hours after injection and then measured using a biotin label–based rat antibody array, which can detect 90 rat proteins (AAR-BLM-1; Ray-Biotech, Norcross, GA). In brief, synovial fluid was first dialyzed with dialysis buffer at 4 °C. Proteins of the dialyzed synovial fluid were measured using a BCA kit (Pierce, Rockford, IL). The samples were labeled with biotin using a labeling reagent from the kit, and samples were incubated with membranes overnight at 4 °C. After being washed, the membranes were incubated with horseradish peroxidase–conjugated streptavidin for 2 hours. Finally, the samples were detected using a Bio-Rad Scanner (Richmond, CA), and the images were analyzed using the RayBio analysis tool.

***Statistical analysis.*** In each experiment, the samples were analyzed in triplicate. Three independent experiments were performed, each with different cartilage preparations of MIA rats. The statistical significance of the differences between groups was calculated using analysis of variance. The results of the same group were evaluated using Student's *t*-test. *P* < 0.05 indicates statistical significance. All data are presented as the mean ± SD.

**SUPPLEMENTARY MATERIAL** **Figure S1.** Immunnohistochemical assessment of the cartilage in MIA rats with miR-101 mimic and inhibitor treatment. **Figure S2.** Immunnohistochemical assessment of the cartilage in normal rats after miR-101 treatment. **Figure S3.** Potential target sites of *Pthlh* and miR-101 predicted by databases. **Table S1.** Identified genes by microarray data analysis. **Table S2.** The changes of cytokines in the synovial fluid. **Table S3.** Rat specific qPCR primer pair sequences.
