# Supplementary material for: Silencing of miR-101 Prevents Cartilage Degradation by Regulating Extracellular Matrix–related Genes in a Rat Model of Osteoarthritis
Source: Mol Ther. 2015 May 26;23(8):1331–40. doi: 10.1038/mt.2015.61 (PMC4817865; doi:10.1038/mt.2015.61)
Supplement: Supplementary Figures and Tables [file mt201561x1.pdf]

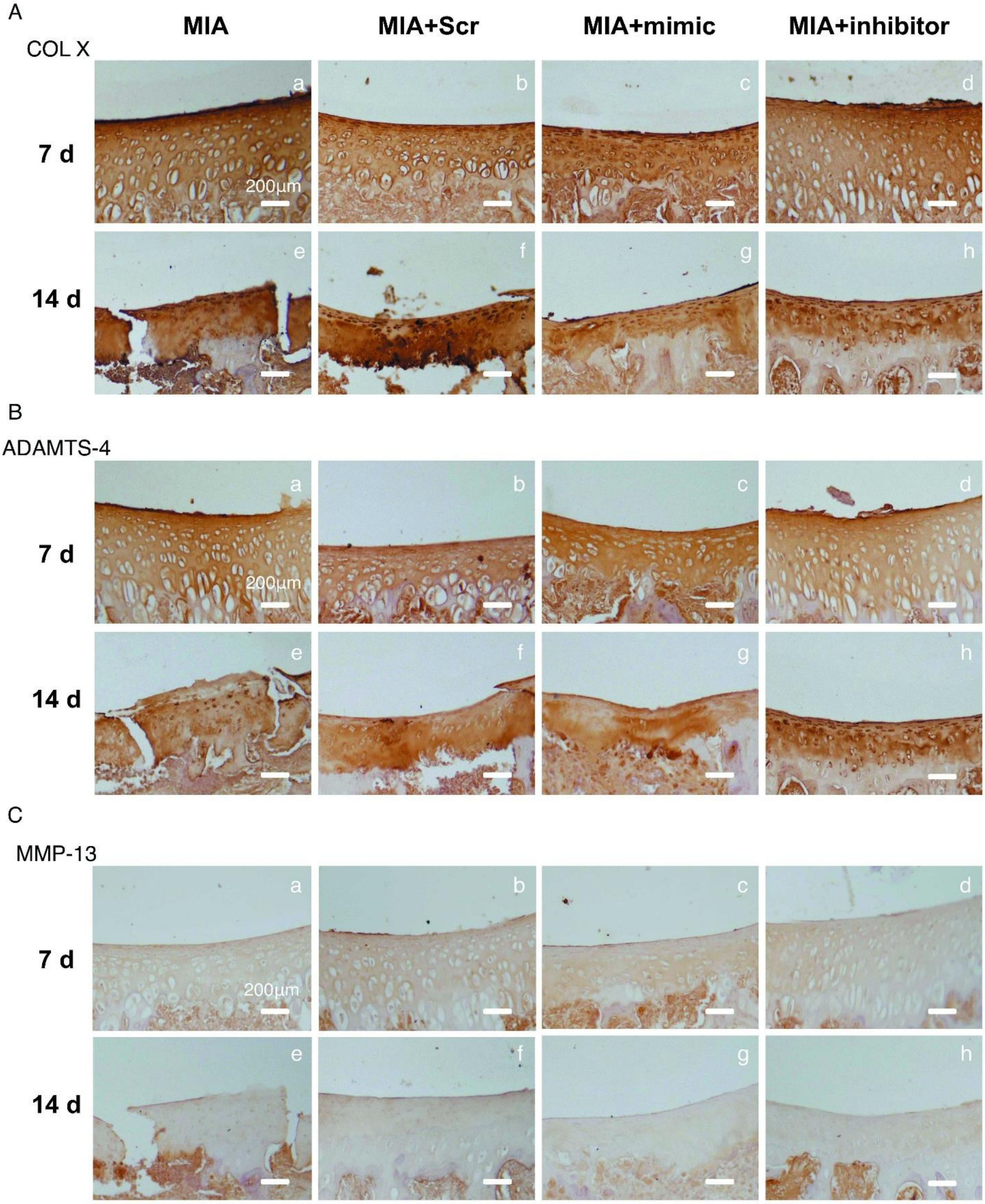

Figure S1. Immunohistochemical assessment of the cartilage in MIA rats with miR-101 mimic and inhibitor treatment. Images in this figure are the representative images of each group. (A) Collagen type X staining images of each group. (B) ADAMTS-4 staining images of each group. (C) IHC staining of MMP-13 in each group. Original magnification is 10×. Bars = 200 μm.

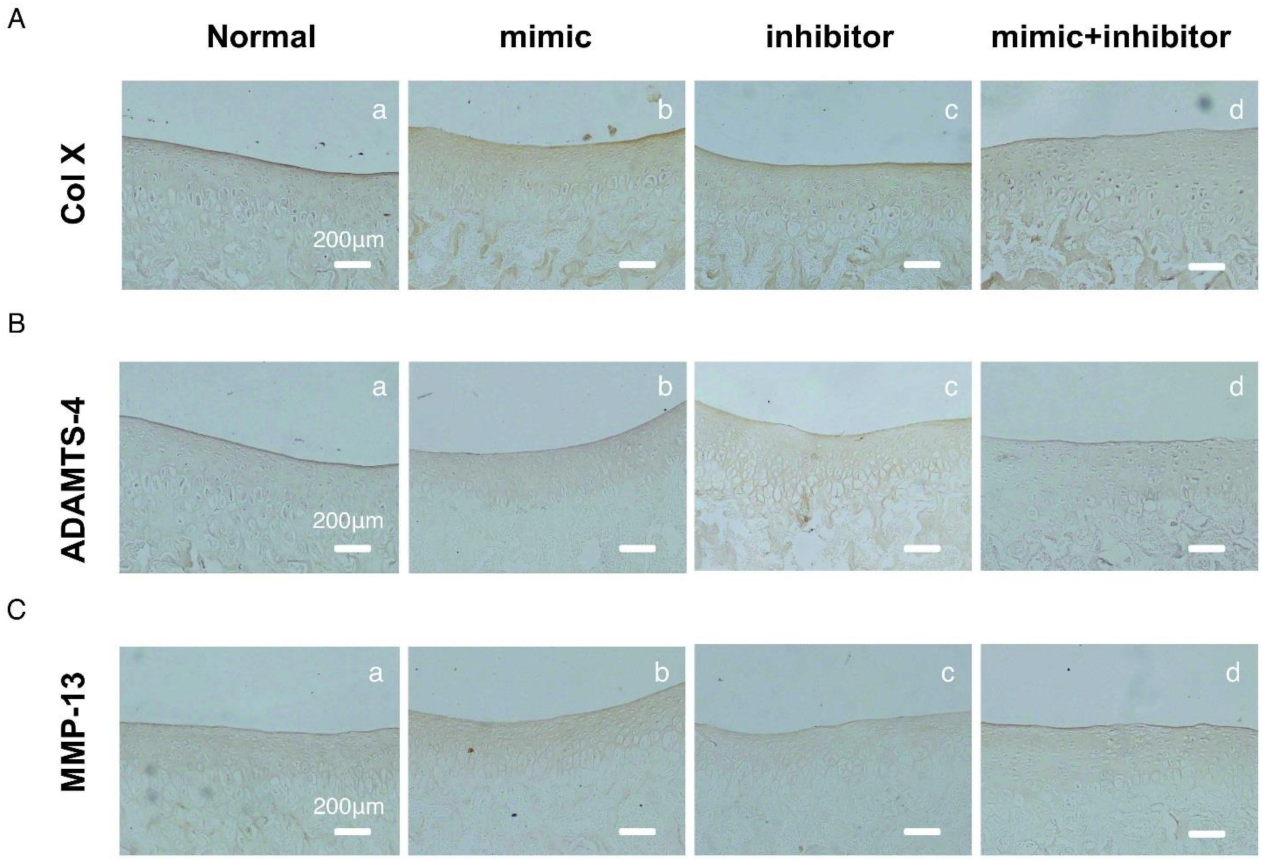

Figure S 2. Immunnohistochemical assessment of the cartilage in normal rats after miR-101 treatment. Images in this figure are the representative images of each group. (A) Collagen type X staining images of each group. (B) ADAMTS-4 staining images of each group. (C) IHC staining of MMP-13 in each group. Original magnification is 10×. Bars = 200 μm.

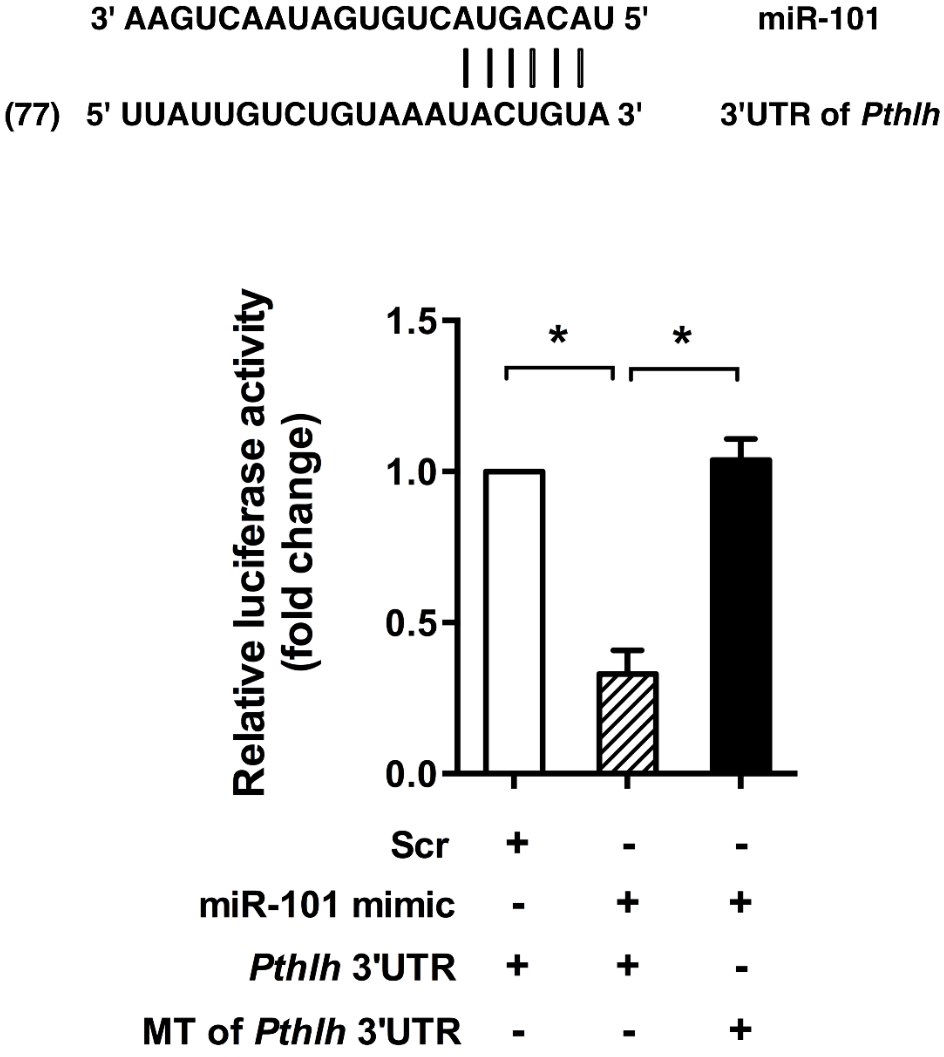

Figure S 3. Potential target sites of *Pthlh* and miR-101 predicted by databases.

The target relationship between miR-101and *Pthlh* were predicted by Pictar (pictar.mdc-berlin.de) and miRanda (http://www.microran.org/microrna/getDownloads.do). Sequences were also acquired according to the above two databases. Luciferase activity of the *Pthlh* 3'UTR reporter was analyzed in HeLa cells. miR-101 mimic were co-transfected with the *Pthlh* 3' UTR or mutant vector. Scrambled 22 nt mimic (Scr) was used as a negative control; n= 3,*P< 0.05 versus Scr group. MT refers to mutant *Pthlh* 3'UTR reporter.

Table S1. Identified genes by microarray data analysis

genes（M vs S）	fold change	M vs S
Angptl4	3.91973	UP
Il6	3.62342	UP
Oasl	3.46417	UP
Ccl20	3.30794	UP
Rgs1	3.24835	UP
Olr1	3.24177	UP
Mir223	3.13017	UP
Ptx3	3.06056	UP
Oas1k	3.00934	UP
RGD1566085	2.44971	UP
LOC314509	2.98175	UP
Nqo1	2.94814	UP
Myoc	2.34801	UP
Adamts5	2.78491	UP
Sh2d1b	2.66223	UP
Amigo2	2.63353	UP
Olr30	2.61956	UP
Fcer1g	2.55208	UP
Ccr5	2.51019	UP
Medag	2.50728	UP
Ier3	2.4667	UP
Nabp1	2.46363	UP
Il1r2	2.46326	UP
Cyp4f18	2.46155	UP
Siglec8	2.4044	UP
LOC501738	2.39456	UP
Tlr7	2.39026	UP
Lilrc2	2.37922	UP
Slc15a3	2.37024	UP
Fgr	2.34745	UP
Cd2	2.32346	UP
Cxcl16	2.32257	UP
RGD1565374	2.31437	UP
LOC100360575	2.31035	UP
Ccr1	2.25846	UP
Ebi3	2.25684	UP
LOC681182	2.25246	UP

Alox5ap	2.25233	UP
Itgal	2.22591	UP
Prdx5	2.21602	UP
Lrg1	2.21381	UP
Ms4a12	2.19465	UP
Itgam	2.18658	UP
Fpr1	2.18538	UP
Myo1f	2.18473	UP
Pik3r5	2.17365	UP
RT1-Bb	2.17272	UP
Fcnb	2.15941	UP
LOC363715	2.15019	UP
Adamts1	2.14569	UP
Naip5	2.14035	UP
Gpr65	2.13834	UP
Wisp2	2.12592	UP
Clec5a	2.11656	UP
Mgst1	2.1143	UP
Itgb7	2.11324	UP
Plin2	2.11245	UP
Fcgr3a	2.10334	UP
Ncf1	2.09223	UP
Ltbp2	2.0906	UP
Postn	2.08821	UP
Lgals3	2.08474	UP
Klra1	2.0781	UP
Folr2	2.07739	UP
Arrdc3	2.07483	UP
Cd302	2.07416	UP
Saa4	2.0713	UP
Ptgr1	2.06391	UP
Errfi1	2.05797	UP
Nkg7	2.04561	UP
Prok2	2.04076	UP
Olr1511	2.03561	UP
Tyrobp	2.02857	UP
Ins1	2.02505	UP
Zfp36	2.01754	UP
Slamf9	2.01157	UP
Gda	2.00718	UP

Dusp1	2.00522	UP
Tmem154	2.00438	UP
LOC367516	2.21652	UP
LOC100366216	2.12194	UP
Ly49si1	2.09969	UP
Serpinb1a	2.09179	UP
Siglec1	2.02131	UP
Tnmd	-5.24593	DOWN
Sfrp5	-2.83915	DOWN
Fbp2	-2.45555	DOWN
Cyp27b1	-2.41666	DOWN
Myl3	-2.40079	DOWN
Kera	-2.38926	DOWN
Plac9	-2.37014	DOWN
Itgbl1	-2.34234	DOWN
Col14a1	-2.3363	DOWN
Fndc1	-2.28807	DOWN
Pthlh	-2.2802	DOWN
U5a	-2.2633	DOWN
Cilp2	-2.23397	DOWN
Sema3a	-2.13474	DOWN
Agtr2	-2.12868	DOWN
Grem1	-2.11623	DOWN
Asb4	-2.11471	DOWN
Clec11a	-2.07783	DOWN
Pdgfrl	-2.04463	DOWN
Col6a6	-2.04143	DOWN
Pon3	-2.00815	DOWN

Table S2. The changes of cytokines in the synovial fluid

cytokines	mimic vs Scr	inhibitor vs Scr
ACTH	7.15±1.34	1.66±0.36
Adiponectin/Acrp30	1.53±0.09	1.05±0.13
AMPK alpha 1	2.91±1.08	1.15±0.27
CINC-3	1.75±0.21	1.18±0.18
CINC-2 alpha/beta	1.61±0.32	1.16±0.21
csk	4.78±1.63	1.80±1.30
EG-VEGF/PK1	1.56±0.11	1.00±0.01
FGF-BP	1.52±0.14	1.06±0.25
Follostatin-like-1(FSL-1)	1.53±0.13	1.18±0.43
Growth Hormone R	1.39±0.24	0.90±0.01
Growth Hormone	1.94±0.14	1.36±0.23
insulin degrading enzyme	1.76±0.34	1.01±0.13
ICK	1.26±0.07	0.96±0.01
IL-1 alpha	4.36±0.67	0.92±0.15
IL-2	1.72±0.11	1.13±0.28
IL-5	1.53±0.52	0.74±0.28
IL-12/IL-23	1.58±0.19	1.06±0.25
IL-13	4.44±0.01	1.66±0.15
inuslin	4.13±0.72	1.51±0.05
IP-10	3.65±0.51	0.83±0.30
MDC	1.65±0.35	1.20±0.23
Neuropilin-2	2.27±0.32	1.57±0.02
Prolactin-R	1.89±0.21	1.19±0.27
RELM beta	2.37±0.42	1.64±0.63
Resistin	2.05±0.16	0.55±0.21
TGF-beta2	3.80±1.54	0.46±0.11
TGF-beta3	7.89±0.34	0.86±0.76
Thrombospondin	3.39±0.67	0.23±0.04
TIMP-3	2.29±0.19	1.53±0.13
Ubiquitin	1.86±0.19	1.03±0.02
VEGF	1.55±0.02	1.14±0.17
IL-10	4.89±0.07	2.52±0.86
Mip-1	6.34±0.32	0.69±0.42
PDGF-AA	1.92±0.01	0.82±0.09
TIMP-2	9.27±1.44	0.88±0.11
CCR4	6.77±2.86	0.74±0.49
CD106	5.29±0.07	1.29±0.05

Fas/TNFRSF6	4.37±0.93	1.34±1.00
Hepassocin	4.35±0.82	0.11±0.01
MIP-2	3.92±1.01	0.62±0.04
MMP-2	2.78±0.80	1.06±0.15

Table S3. Rat specific qPCR primer pair sequences

Target	Forward primer sequence	Reverse primer sequence
ADAMTS-1	5’-GCCTACATGGTCACATCATTCCT-3’	5’-TGGCGGTTGGCATCGTA-3’
ADAMTS-5	5’-CACGACCCTCAAGAACTTTTGC-3’	5’-TCACATGAATGATGCCCACATAA-3’
IL-6	5’-TGAGAAAAGAGTTGTGCAATGG-3’	5’-GCATCATCGCTGTTCATACAAT-3’
PTHLH	5’-GCCAAGGCTAATCCAATTATTAC-3’	5’-TCAGAACTCATCAAAATGAACCAC-3’
POSTN	5’-AACCAAGGACCTGAAACACG-3’	5’-CAACACCATTTGTGGCAATC-3’
ITGA1	5’-TCCAGCTTAAATCTTACCATACG-3’	5’-ATTAACAGCAGGAGTCCCGCAA-3’
SOX9	5’-AGGAAGCTGGCAGACCAGTA-3’	5’-ACGAAGGGTCTCTTCTCGCT-3’
18S	5’-GTAACCCGTTGAACCCCATT-3’	5’-CCATCCAATCGGTAGTAGCG-3’
